# A new animal model of spontaneous autoimmune peripheral polyneuropathy: implications for Guillain-Barré syndrome

**DOI:** 10.1186/2051-5960-2-5

**Published:** 2014-01-08

**Authors:** Mu Yang, Anthony Rainone, Xiang Qun Shi, Sylvie Fournier, Ji Zhang

**Affiliations:** 1 Department of Neurology and Neurosurgery, McGill University, Montreal, QC H3A 2B4, Canada; 2 Alan Edwards Centre for Research on Pain, McGill University, Montreal, QC H3A 0G1, Canada; 3 Department of Microbiology and Immunology, McGill University, Montreal, QC H3A 2B4, Canada; 4 Faculty of Dentistry, McGill University, Montreal, QC H3A 0C7, Canada

**Keywords:** Autoimmune response, Demyelination, T cell co-stimulation, CD8^+^ T cells, Animal model

## Abstract

**Background:**

Spontaneous autoimmune peripheral neuropathy including Guillain-Barré Syndrome (GBS) represents as one of the serious emergencies in neurology. Although pathological changes have been well documented, molecular and cellular mechanisms of GBS are still under-explored, partially due to short of appropriate animal models. The field lacks of spontaneous and translatable models for mechanistic investigations. As GBS is preceded often by viral or bacterial infection, a condition can enhance co-stimulatory activity; we sought to investigate the critical role of T cell co-stimulation in this autoimmune disease.

**Results:**

Our previous study reported that transgene-derived constitutive expression of co-stimulator B7.2 on antigen presenting cells of the nervous tissues drove spontaneous neurological disorders. Depletion of CD4^+^ T cells in L31 mice accelerated the onset and increased the prevalence of the disease. In the current study, we further demonstrated that L31/CD4^-/-^ mice exhibited both motor and sensory deficits, including weakness and paresis of limbs, numbness to mechanical stimuli and hypersensitivity to thermal stimulation. Pathological changes were characterized by massive infiltration of macrophages and CD8^+^ T cells, demyelination and axonal damage in peripheral nerves, while changes in spinal cords could be secondary to the PNS damage. In symptomatic L31/CD4^-/-^ mice, the disruption of the blood neural barriers was observed mainly in peripheral nerves. Interestingly, the infiltration of immune cells was initiated in pre-symptomatic L31/CD4^-/-^ mice, prior to the disease onset, in the DRG and spinal roots where the blood nerve barrier is virtually absent.

**Conclusions:**

L31/CD4^-/-^ mice mimic most parts of clinical and pathological signatures of GBS in human; thus providing an unconventional opportunity to experimentally explore the critical events that lead to spontaneous, autoimmune demyelinating disease of the peripheral nervous system.

## Background

Guillain-Barré Syndrome (GBS) is an acute immune-mediated polyradiculoneuropathy comprising a broad spectrum of clinical variants, including acute inflammatory demyelinating polyradiculoneuropathy (AIDP), acute motor axonal neuropathy (AMAN), acute motor and sensory axonal neuropathy (AMSAN) and the Miller-Fisher Syndrome (MFS). In its typical form, GBS causes rapidly progressive diffuse weakness or paralysis of the four limbs, sensory loss or pain and areflexia [1]. The sudden and unexpected onset of paralysis is devastating for patients and represents one of the serious emergencies in neurology. The pathological substrate for GBS has been well established with immune cell infiltration, demyelination with or without axonal damage in the peripheral nervous system (PNS), nevertheless molecular and cellular mechanisms have not been fully understood. Although experimental allergic neuritis (EAN) induced by immunization with peripheral nerve proteins or adoptive transfer of sensitized T cells [2,3] has provided valuable information, it has been criticized for its artificial manipulation [4]. Only one animal model with spontaneous autoimmune peripheral polyneuropathy has been reported in non-obese-diabetic (NOD) mice [5].

T cell costimulation has a central role in autoimmunity [6]. Infection could promote autoimmunity via inducing the co-stimulatory activity in antigen presenting cells(APCs), which are required to induce the expansion of antigen-reactive T cells, migration of T cells to the site of inflammation and the production of inflammatory mediators [7,8]. Expression of co-stimulatory molecule B7 was enhanced in the CNS of MS patients and in inflamed nerves of GBS patients [9,10]. Targeting B7.2/CD28 signaling pathway has been fairly common in experimental studies of autoimmune diseases [11].

We have previously reported that constitutive expression of B7.2 on APCs in the nervous system (L31 mice) led to neurological dysfunction with spontaneous demyelination and immune cell infiltration in the spinal cords and spinal roots [12]. Both T cells and the expression of B7.2 on spinal cord microglia were required for the development of the disease [12]. A dramatic skewing toward CD8^+^ T cells in the nervous system suggests a critical role of CD8^+^ T cells in the pathogenesis [12], which has been further supported by the fact that depletion of CD4^+^ T cells in L31 mice accelerated the onset and increased the prevalence of the disease [13].

In the current study, we used L31 mice on a genetic background where CD4^+^ T cells were depleted (CD4^-/-^) to investigate whether constitutive expression of B7.2 could also drive spontaneous demyelination in the PNS and to seek insights for GBS in human. We observed that in addition to motor deficits as reported previously, L31/CD4^-/-^ mice also developed sensory disorders, including numbness to mechanical stimuli and hypersensitivity to thermal stimulation. Severe demyelination, massive infiltration of macrophages, CD8^+^ T cells, and axonal damage were found predominantly in the PNS, while changes in the spinal cords could be secondary to the PNS damage. The data revealed that L31/CD4^-/-^ mice mimic most parts of clinical and pathological aspects of GBS, suggesting constitutive expression of B7.2 in APCs could be a new and useful animal model of GBS for mechanistic studies.

## Methods

### Animals

L31 mice with constitutive expression of co-stimulatory B7.2/CD86 were generated with MHC I promoter and Igμ enhancer [14]. L31/CD4^-/-^ mice have been described previously [13] and were maintained by successive backcross to C57BL/6 mice for more than 7 generations. C57BL/6 mice bred and housed in the same facility were used as controls. All procedures were in accordance with the guidelines of the Canadian Council on Animal Care, and approved by the animal care committee of McGill University. In total, 49 L31/CD4^-/-^ and 18 C57BL/6 mice (controls), aged at 2-6 months, were included in the study. 29 L31/CD4^-/-^ symptomatic mice and 6 C57BL/6 mice were monitored for their clinical scores and behavioral responses. 20 L31/CD4^-/-^ mice were euthanized before disease onset, as pre-symptomatic mice for tissue collection.

### Instruments and reagents

All instruments and reagents have been used in the study were listed in Additional file 1: Table S1.

### Assessment of neurological disorders

All mice included in the current study had their disease onset between 2-4 months after birth. Animals whose clinical score ≥4 were excluded from sensory tests.

*Clinical scores for motor deficits:* 0 = normal; 1 = reduced tonus of tail and/or limp tail; 2 = weakness of one hind limb, staying in clasping or outstretching position when lifted by the tail; 3 = weakness of two hind limbs, both staying in clasping or outstretching position when lifted by the tail; 4 = paresis or splaying of one hind limb; 5 = paresis or splaying of two hind limbs; 6 = moribund or death. L31/CD4^-/-^ transgenic mice with clinical score at 0 were defined as pre-symptomatic mice.

*Rotarod assay* was used to assess mouse motor coordination. The task included a speed ramp from 0 to 30 rpm over 60 s, followed by an additional 240 s at the maximal speed. The time that each animal walked on the rod before falling was recorded. A decrease in walking time is suggestive of motor impairment.

*von Frey Test* was performed to test paw sensitivity to mechanical stimuli. Calibrated monofilaments were applied to the plantar surface of the hindpaw and the 50% threshold to withdraw was calculated as previously described [15]. A decrease in threshold suggests the development of mechanical allodynia.

*Acetone Test* was used to evaluate sensitivity to cold stimuli. Total duration of acetone evoked behaviors (flinching, licking or biting) was measured for 1 minute after one drop of acetone (~25 μl) was applied to the plantar surface of the hindpaw. An increase of withdrawal duration indicates the cold allodynia.

*Hot plate* (55°C) was used as an unpleasant sensory heat stimulus to measure pain response. The latency to paw-licking, squeaking, or distressful behavior was measured. A decrease in latency suggests the development of heat hypersensitivity.

### Tissue preparation

*For histological studies* Lumbar spinal cords, L4-L6 DRGs, dorsal roots and sciatic nerves were collected with transcardiac perfusion of 4% paraformaldehyde or cold saline. L4-L6 DRG, roots and sciatic nerves were cut either longitudinally into 12 μm sections or at 5 μm cross-sections with a cryostat. Lumbar spinal cords were cut into 25 μm-thick sections using microtome.

*For flow cytometric studies* Mice were deeply anaesthetized with isoflurane. Approximately 1 cm long of lumbar spinal cord and 2 cm long of sciatic nerves were collected. Samples were diced into very small pieces in RPMI-1640 medium with streptomycine/penicilline and 10% fetal bovine serum.

### Immunohistochemistry and image acquisition

Regular immunofluorescent staining was performed as previously described [16]. Tissues were incubated with primary antibodies overnight at 4°C, followed by fluorochrome-conjugated secondary antibodies for 60 min at room temperature, and then counterstained with marker for nucleus 4′, 6-Diamidino-2-phenylindole dihydrochloride (DAPI) and/or FluoroMyelin. Details of all antibodies used in the study were listed in Table 1. Images were acquired using an Olympus BX51 microscope equipped with a color digital camera or Olympus confocal laser-scanning microscope.

**Table 1:** List of antibodies used in the study

**Antibody/dye**	**Specificity**	**Immunogen/clone**	**Dilution**	**Source**	**Catalog#**
*Primary antibodies for IHC*					
Rabbit anti ionized calcium-binding adaptor molecula 1 (Iba-1)	17 kDa EF hand protein expressed on macrophages and microglia	Synthetic peptide of C-terminus Iba-1 N’-PTGPPAKKAISELP-C’	1:1000	Wako	019-19741
Rat anti mouse CD86	Mouse B7.2	LPS-activated CBA/Ca mouse splenic B cells	1:600	Biolegend	105010
Rabbit anti protein gene Product 9.5 (PGP9.5)	All mammalian species, PGP9.5 expressed on neuronal cell bodies and axons in central and peripheral neural system	Human PGP 9.5 Protein purified from pathogen-free human brain	1:800	Ultraclone	RA95101
Rabbit anti human activating transcription Factor 3 (ATF3)	Mouse, rat and human, ATF3 expressed on damaged neurons	A peptide mapping at the C-terminus of human ATF-3	1:2000	Santa cruz	SC188
Rat anti mouse CD8 alpha	Mouse CD8 alpha	Mouse thymus/spleen cells	1:2000 (fresh frozen tissues with short fixation)	BD pharmingen	550281
Rabbit anti glucose transporters-1 (Glut1)	Mouse, rat and human, Glut1expressed on endothelial cells and perinurium	Synthetic peptide corresponding amino acids with the C-terminus of human GLUT-1 coupled to KLH (C-ELFHPLGADSQV)	1:6000	Merck millipore	07-1401
*Secondary antibodies for IHC*					
Alexa fluor 594 goat anti-rabbit IgG	IgG heavy chains and all classes of IgG light chains from rabbit		1:500	Molecular probes (Invitrogen)	A11037
Alexa fluor 488 goat anti-rabbit IgG	IgG heavy chains and all classes of IgG light chains from rabbit		1:500	Molecular probes (Invitrogen)	A11034
Alexa fluor 594 rabbit anti-rat IgG	IgG heavy chains and all classes of IgG light chains from rat		1:500	Molecular probes (Invitrogen)	A11007
Alexa fluor 488 rabbit anti-rat IgG	IgG heavy chains and all classes of IgG light chains from rat		1:500	Molecular probes (Invitrogen)	A11006
*Dye*					
4′,6-diamidino-2-phenylindole dihydrochloride (DAPI)			1:15,000	Sigma-aldrch	D9542
Fluoro myelin red fluorescent myelin stain			1:300	Molecular probes (Invitrogen)	F34652
*Flow cytometric antibodies*					
FITC rat anti-mouse CD11b	Mouse CD11b^+^ cells	C57BL/10 splenic T cells and concanavalin A-activated C57BL/10 splenocytes	1:50	BD pharmingen	553310
Rat anti-mouse CD45 APC	Mouse CD45^+^ cells	Clone 30‒F11	1:50	eBioscience	17-0451-82
Rat anti-mouse CD8 alpha PerCP-Cy5.5	Mouse CD8^+^ cells	Clone 53-6.7	1:50	eBioscience	45-0081-82
Rat anti-mouse CD86 (B7.2) PE	Mouse B7.2^+^ cells	Clone GL1	1:50	eBioscience	12-0862-82

### Flow cytometric analysis

Single-cell suspensions and blocking were prepared as described previously [16]. Samples were then stained with specific fluorochrome-conjugated antibodies for 25 min at 4°C. Staining specificity was identified by omitting antibodies, and correlation of spectral overlap was done by using negative and positive compensation beads. Cellular events were acquired using a LSR Fortessa flow cytometer and data was analyzed using Flow Jo software. Detail information of antibodies used here was listed in Table 1.

### Evaluation of blood-neural barrier permeability

Blood nerve barrier (BNB), blood spinal cord barrier (BSCB) and blood brain barrier (BBB) permeability was assessed using a micromolecular tracer sodium fluorescein (NaFlu). The protocol was adopted and modified from previous studies [17]. Briefly, NaFlu was administrated intravenously (10%; 2 ml/kg) and allowed to circulate for 30 min. Then, mice were transcardially perfused with cold saline for 10 min to remove intravascular NaFlu. The choroid plexuse, meninge and epineurium were removed before nervous tissues were dissected into different regions: brain, cervical and lumbar spinal cords, and sciatic nerves. Following tissue homogenization, the concentration of NaFlu in supernatant was measured with spectrophotofluorometer. NaFlu was expressed as g/g of tissue.

### Statistical analysis

Data is presented as means ± SEM. Unpaired *t*-test was used to determine significance between groups. A value of P < 0.05 was accepted as statistically significant.

## Results

### Constitutive expression of B7.2 in APCs primed resident macrophages in sciatic nerves and microglia in spinal cords

**Figure 1:**
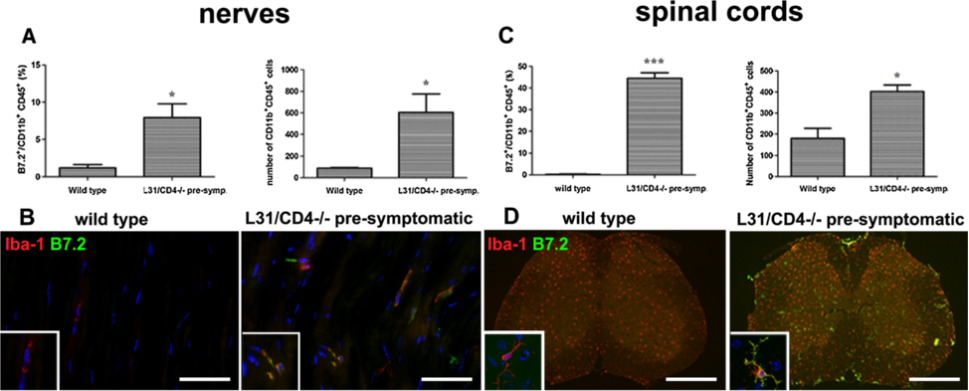
**Transgene-derived B7.2 constitutive expression primed macrophages and microglia in the nervous tissues.** The number of CD11b^+^ CD45^+^ macrophages in the sciatic nerves and microglia in the spinal cord, and the expression of B7.2 among these targeted cells was quantified using fluorescent-activated cell sorting (FACS) analysis. The histogram represents the FACS analysis obtained from 3 animals per group. Percentage of either macrophages **(A)** or microglia **(C)** expressing B7.2 is increased in L31/CD4^-/-^ mice, compared with wild type animals. The absolute number of macrophages in the nerves and microglia in the spinal cord is also higher in L31/CD4^-/-^ mice than that of wild type mice **(A, C)** (*: p < 0.05, ***: p < 0.001). Representative examples of immunohistochemistry analysis of macrophages in the sciatic nerves and microglia in the spinal cords were depicted in **(B)** and **(D)**. Note that in L31 /CD4^-/-^ mice, many Iba-1^+^ macrophages and microglia were colocalized with B7.2 which was almost undetectable in wild type mice. Scale bars: B, 50 μm; D, 500 μm.

B7.2 expression was verified by flow cytometry on macrophages and microglia, isolated from sciatic nerves and lumbar spinal cords (Additional file 2: Figure S1). Increased expression of B7.2 was detected in macrophages of L31/CD4^-/-^ mouse sciatic nerves (Figure 1A-B), which was derived most likely from B7.2 transgene, as seen in spinal microglia, resident macrophages in the CNS [13]. It appears that B7.2 transgene primed macrophages by increasing macrophage cell density (Figure 1A). Immunohistochemistry analysis confirmed that L31/CD4^-/-^ mice had more Iba-1^+^ macrophages in sciatic nerves, and these were elongated resident macrophages [18]. Many of them were double-labelled by Iba-1 and B7.2 (Figure 1B). Similar changes were detected in the spinal cords (Figure 1C-D). Pre-symptomatic L31/CD4^-/-^ mice had more CD11b^+^ CD45^+^ microglia than wild type mice (Figure 1C). Many of them expressed B7.2, while the expression of B7.2 was almost undetectable in wild type mice (Figure 1C-D).

### L31/CD4^-/-^ mice developed spontaneous motor and sensory neurological disorders

**Figure 2:**
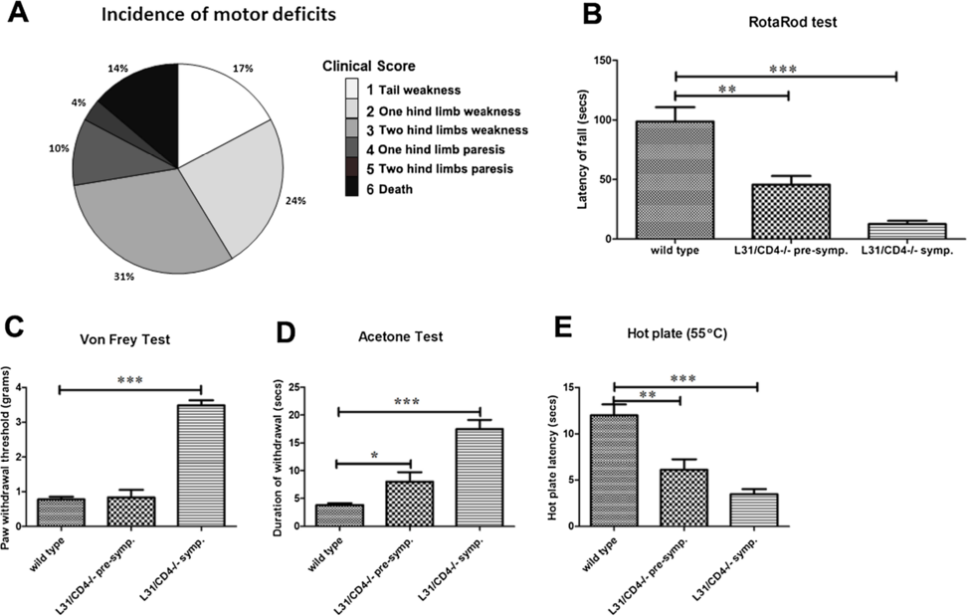
**L31/CD4**^**-/- **^**mice developed spontaneous motor and sensory neurological disorders.** The incidence of motor deficits in L31/CD4^-/-^ mice evaluated with established clinical scores was presented in **(A)**. Rotarod test was performed to examine coordination ability. Compared with wild type mice, the latency of fall dropped in both pre-symptomatic and symptomatic L31 mice **(B)** (n = 6/group, **: p < 0.01, ***: p < 0.001), indicative of motor impairment. Pain responses to mechanical, cold and heat stimuli were assessed in wild type, pre-symptomatic and symptomatic L31/CD4^-/-^ mice. Animals that had their clinical score ≥4 were excluded from sensory tests. Only symptomatic L31/CD4^-/-^ mice demonstrated numbness to von Frey hair stimulation **(C)** (n = 6/group, ***: p < 0.001). However, thermal hypersensitivity was observed in both pre-symptomatic and symptomatic L31 mice, as the duration of withdrawal in acetone test increased **(D)** (n = 6/group, *: p < 0.05, ***: p < 0.001), and the hotplate latency decreased **(E)** (n = 6/group, **: p < 0.01, ***: p < 0.001).

Almost all L31/CD4^-/-^ mice developed motor deficits (Additional file 3: Video 1 and 2, Figure 2A) within 4 months after birth. Majority (55%) demonstrated hind limb weakness (score 2-3), while walking was not altered significantly. However, 14% mice had difficulties walking properly. One or two hind limbs were found with paresis, in a splaying position (score 4-5). Whatever the severity of the illness, the symptoms appeared suddenly, reaching a maximum within 3-7 days. Most animals, after the appearance of neurological signs, remained stable. Among 29 L31/CD4^-/-^ symptomatic mice monitored for behavior, 4 died of illness (14%). No apparent recovery was found within the observation period (maximum 2 months following disease onset).

Poor coordination was observed in both pre-symptomatic (clinical score = 0, ≥ 2 months old) and symptomatic L31/CD4^-/-^ mice (clinical score ≥1). The latency of fall in Rotarod test dropped from 98.60 ± 12.01 sec in wild type mice to 45.50 ± 7.27 sec in pre-symptomatic mice. Symptomatic mice could only stand on the running rod for 12.5 ± 2.72 sec (Figure 2B).

L31/CD4^-/-^ mice also developed sensory disorders. Symptomatic animals exhibited numbness to von Frey hair as paw withdrawal thresholds reached at 3.48 ± 0.15 g, while wild type and pre-symptomatic mice responded at 0.78 ± 0.07 g and 0.83 ± 0.21 g, respectively (Figure 2C). However, they were hypersensitive to cold and heat stimulation. Duration of withdrawal in acetone test was increased (Figure 2D) and the hot plate latency was decreased (Figure 2E) in symptomatic mice. Interestingly, thermal hypersensitivity appeared prior to motor dysfunctions, since alteration in response to cold and heat stimulation was observed in pre-symptomatic mice (Figure 2D-E).

### Demyelination and axonal damage in peripheral nerves of symptomatic L31/CD4^-/-^ mice

**Figure 3:**
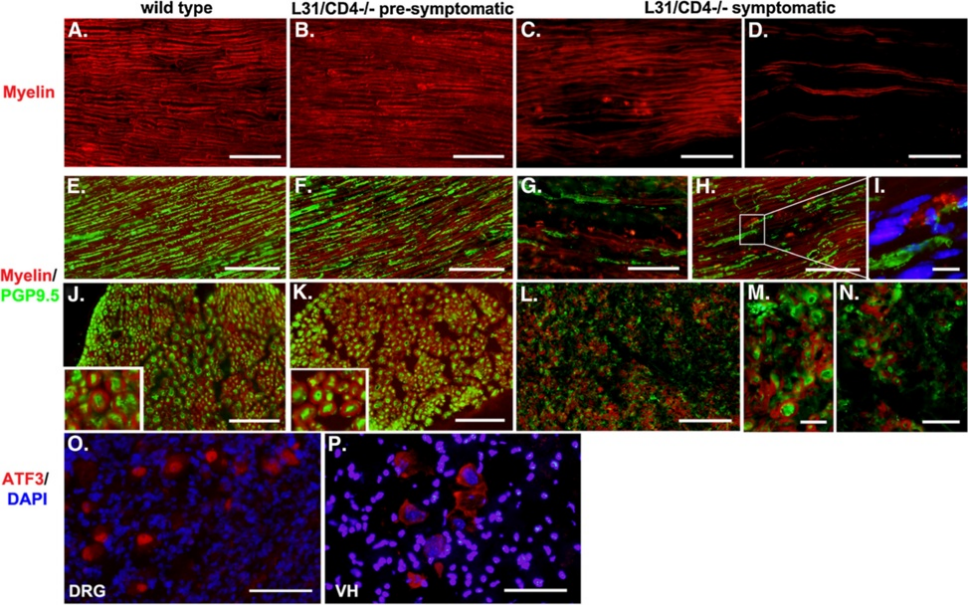
**Demyelination and axonal damage were found in the peripheral nerves of symptomatic L31/CD4**^**-/- **^**mice.** Myelin in the sciatic nerves was stained with FluoroMyelin. Representative illustrations from longitudinal **(A-I)** and cross **(J-N)** sections showed an intact myelin structures in wild type **(A, E, J)** and pre-symptomatic L31 mouse sciatic nerves **(B, F, K)**, and a focal **(C, H)** or diffused, severely damaged myelin **(D, G)** in symptomatic L31 mice. In parallel with demyelination, the axonal damage evidenced by the loss of PGP9.5 staining was detected in symptomatic L31 mice **(G, H, L, M, N)**. Some naked PGP9.5^+^ axonal fibers were found in the area where myelin disappeared or fragmented **(G, H, L, M, N)**. **I**: Myelin labelling was found in a cell-shaped structure containing a DAPI^+^ nucleus, which suggests a macrophage with engulfed myelin fragments. Two naked axonal fibers were located nearby. ATF3 positive signals were found in the cell bodies of both DRG sensory neurons **(O)** and spinal cord ventral horn (VH) motor neurons **(P)**. Scale bar: 50 μm.

Immunohistochemistry studies demonstrated that myelin (Figure 3B, F, K) and axonal structures (Figure 3F, K) in pre-symptomatic mice remained in healthy condition. Same as in wild type mice (Figure 3A, E, J), myelin sheaths in pre-symptomatic mice appeared as wide straight lines in longitudinal sections (Figure 3B) and wrapped around PGP9.5^+^ axons in longitudinal (Figure 3F) and cross sections (Figure 3K). However, focal (Figure 3C) or severe, diffused (Figure 3D) myelin loss was detected in symptomatic L31 CD4^-/-^ mice. Destruction of the myelin in symptomatic mice was most frequently accompanied by axonal damage where the number of PGP9.5^+^ axonal fibers was significantly reduced (Figure 3G, H, L). Nevertheless, some naked PGP9.5^+^ axonal fibers were found in the area where myelin disappeared or fragmented (Figure 3G, H, M, N), indicating demyelination might occur, at least in some area, prior to the axonal damage. Image with high magnification in Figure 3I revealed a cell-shaped myelin labeling with a DAPI^+^ nucleus inside, suggesting presumably it could be a macrophage engulfing myelin debris, while two nude axons were apposed closely. The detection of ATF3, a marker for neuronal degeneration, in DRG sensory neurons (Figure 3O), as well as in spinal cord motor neurons (Figure 3P) suggests that both sensory and motor neurons were affected.

### Massive immune cell infiltration in the sciatic nerves of symptomatic L31/CD4^-/-^ mice

**Figure 4:**
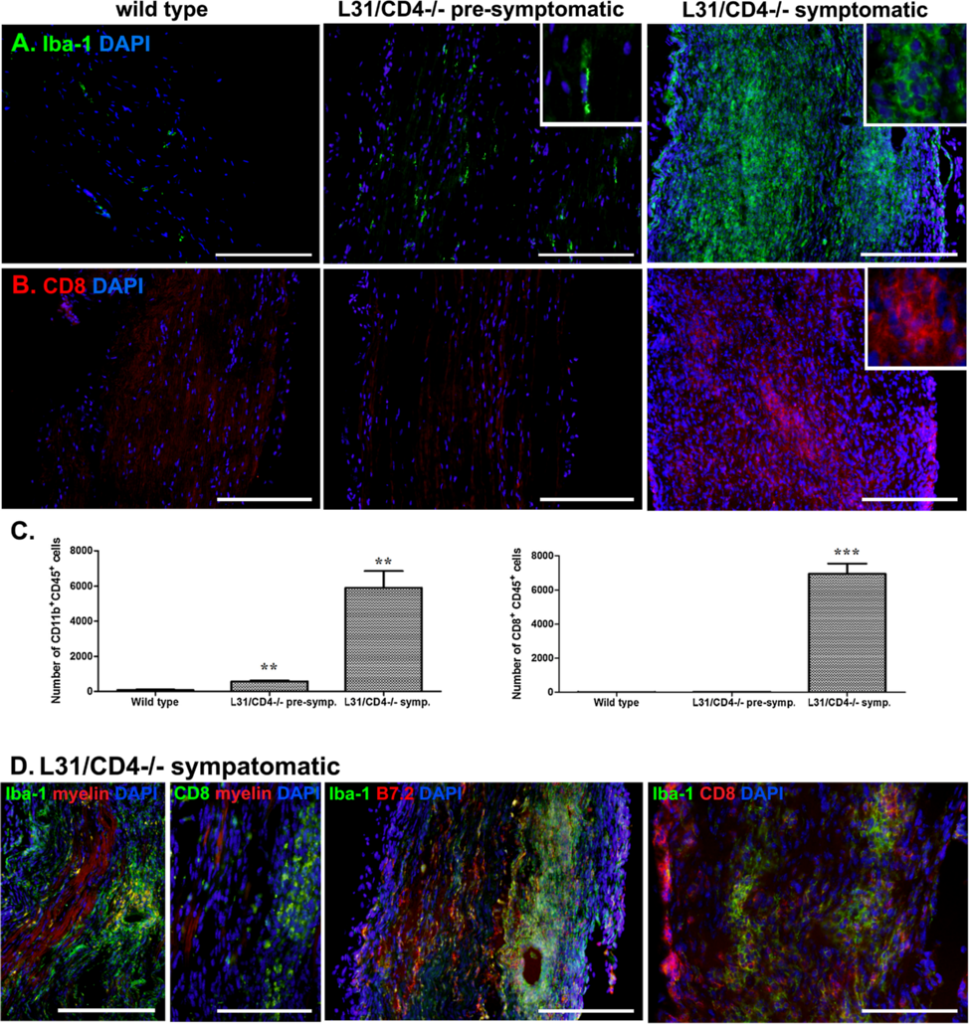
**Massive infiltration of immune cells in sciatic nerves of L31/CD4**^**-/- **^**mice.** Cell infiltration in the diseased sciatic nerves was first evidenced by the significant increase of DAPI labelled cell number **(A-B)**. Immunohistochemistry analysis demonstrated that while there was a slight increase of elongated (insert) Iba-1^+^ cells in the pre-symptomatic L31/CD4^-/-^ mice, nerves from the symptomatic L31/CD4^-/-^ mice were submerged with round-shaped (insert) infiltrated Iba-1^+^ macrophages **(A)**. While there were abundant CD8^+^ T cells found in the sciatic nerves of symptomatic L31 mice, no CD8^+^ T cell infiltration was detected in pre-symptomatic L31/CD4^-/-^ mice **(B)**. Quantitative analysis with FACS (n = 3/group) confirmed the immunohistochemistry observation (**: p < 0.01, ***: p < 0.001) **(C)**. In symptomatic L31 mouse sciatic nerves, infiltrates could either group in small foci **(D-left)** or be distributed diffusely **(A/B-right)**. Fragmented myelin were found within Iba-1^+^macrophages (yellow signals within Iba-1^+^ cells). There were less infiltrates in the area where myelin remained in healthy shape **(D-left)**. Majority of infiltrated Iba-1^+^ macrophages had high levels of B7.2 expression **(D-middle)** and CD8^+^ T cells were in close apposition with Iba-1^+^ macrophages **(D-right)**. Scale bar: 200 μm.

In parallel to the demyelination and axonal damage, there was a massive infiltration of immune cells into the nerves (Figure 4). They consisted of mainly round-shaped Iba-1^+^ macrophages (Figure 4A) and CD8^+^ T cells (Figure 4B). Apart from a slight increase of elongated resident macrophages (Figure 4A, insert in the middle panel), no round-shaped macrophages or CD8^+^ T cells were found in the sciatic nerves of pre-symptomatic mice (Figure 4A-B). Immune cell infiltration was confirmed by quantitative flow cytometry analysis (Figure 4C, Additional file 4: Figure S2). In symptomatic mice (Figure 4D), infiltrates were either grouped in small foci or distributed diffusely. In the vicinity of cellular clusters, myelin sheaths were disintegrated or replaced by oval-shaped vacuoles. Fragmented myelin was found within Iba-1^+^macrophages (yellow signals within Iba-1^+^ cells). There were less infiltrates in areas where myelin remained in healthy-shape (Figure 4D-left). Majority of infiltrated Iba-1^+^ macrophages had high levels of B7.2 expression (Figure 4D-middle) and CD8^+^ T cells were in close apposition with Iba-1^+^ macrophages (Figure 4D-right).

### Limited demyelination and immune cell infiltration in the spinal cords of L31/CD4^-/-^ mice

**Figure 5:**
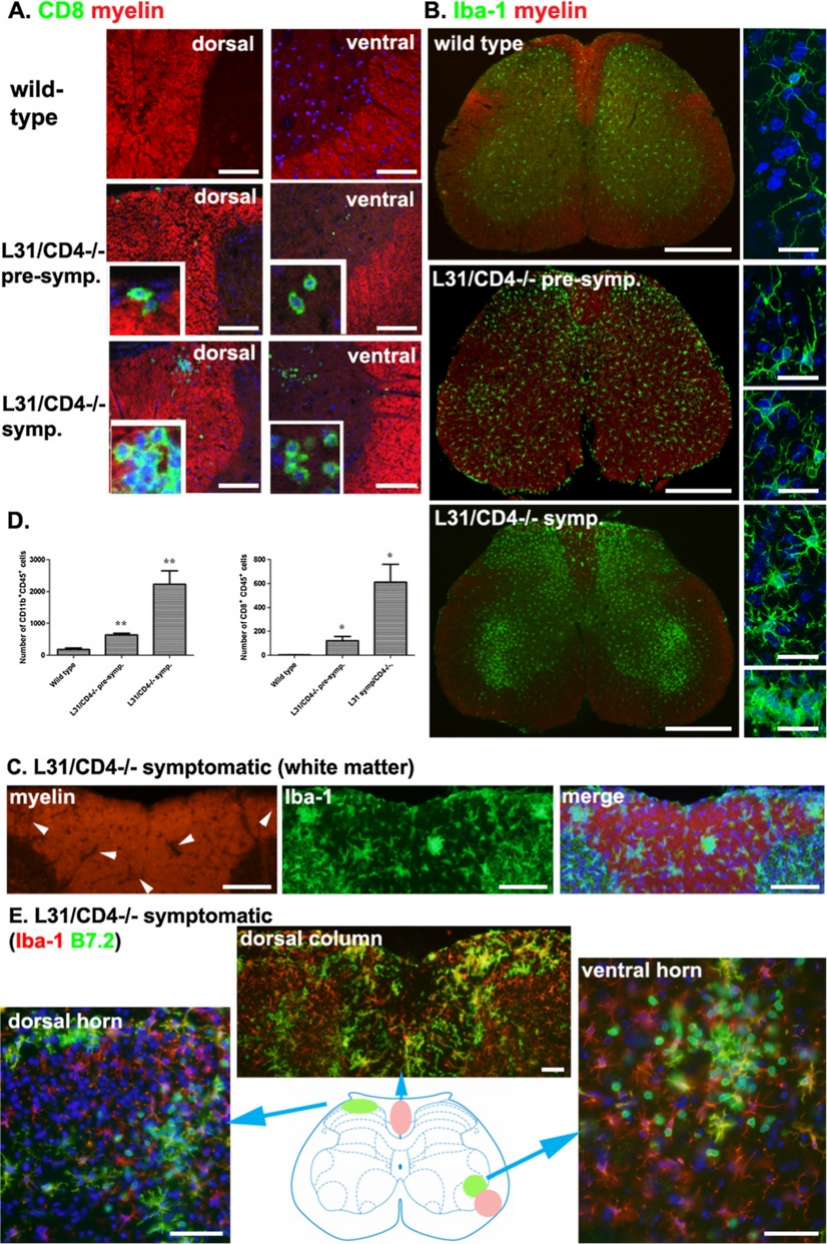
**Limited demyelination and immune cell infiltration was detected in the spinal cords of L31/CD4**^**-/- **^**mice.** Only isolated myelin loss was found in the spinal white matter of symptomatic L31/CD4^-/-^ mice together with few CD8^+^ T cell clusters **(A)**. CD8^+^ T cells were also found in the grey matter of some symptomatic L31/CD4^-/-^ mice, gathered near motor neurons **(A)**. Occasionally very few isolated CD8^+^ T cells were found even in pre-symptomatic L31/CD4^-/-^ mouse spinal cords **(A)**. In symptomatic L31/CD4^-/-^ mice, robust spinal microglia activation was detected, mainly in the grey matter. Iba-1^+^ microglia were clustered at the dorsal and ventral horns **(B)**. Morphological changes were revealed with confocal microscope **(B-left panels)**. In the white matter of diseased spinal cord, some demyelinated spots **(C-arrows)** were found in the dorsal column where Iba-1^+^ microglia gathered **(C)**. Quantitative analysis of CD11b^+^CD45^+^ microglia and CD8^+^ T cells using FACS (n = 3/group) confirmed the immunohistochemistry observation (*: p < 0.05, **: p < 0.01) **(D)**. Increased B7.2 expression was found in symptomatic spinal cords, on ramified Iba-1/B7.2 double labelled microglia, or on round B7.2 single labelled cells, presumably CD8^+^ T cells **(E)**. Scale bars: A, 100 μm; B, left, 500 μm, right, 10 μm; C, 100 μm; E, 50 μm.

Myelin structures in the spinal cords of pre-symptomatic mice remained intact. Very little spotted demyelinating lesions could be found only in severely diseased animals. Even then, they were restricted to the dorsal columns and the areas surrounding ascending spinothalamic tracts (Figure 5A, D and E - pink zone). Clusters of infiltrated CD8^+^ T cells were seen within demyelinated spots or damaged motor neurons (Figure 5A). A few isolated CD8^+^ T cells were also detected in pre-symptomatic mice where myelin architecture was not compromised (Figure 5A). Robust spinal microglia activation was detected in symptomatic mice, predominantly in the grey matter (Figure 5B and 5E-green zone). Iba-1^+^ microglia were clustered at the dorsal horns where central afferents of sensory neurons terminate and in the ventral horns surrounding motor neurons (Figure 5B). The topography and morphological changes are exactly the same as the spinal microglial activation triggered by a physical injury to the sciatic nerve [19], which suggests that microglial activation in the grey matter of symptomatic mice could be a response secondary to the PNS damage. However, in the white matter, activated microglia were found restricted to the zone where myelin was destroyed (Figure 5C, arrows). The increase of CD11b^+^CD45^+^ microglia and CD8^+^ T cells were quantified by flow cytometry (Figure 5D, Additional file 5: Figure S3). Either in grey matter or white matter, activated microglia had high levels of B7.2 expression as many B7.2^+^ cells were colocalized with ramified Iba-1^+^ cells. Due to the incompatibility of the antibodies, CD8 and B7.2 antibodies could not be used on the same tissue section, however presumably round shaped B7.2 single labelled cells were infiltrated CD8^+^ T cells (Figure 5E).

### Disruption of blood nerve barrier in the sciatic nerves of diseased L31/CD4^-/-^ mice

**Figure 6:**
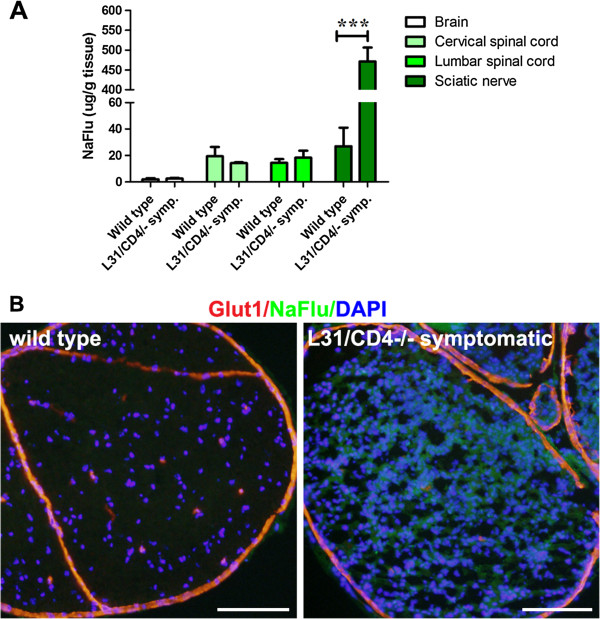
**Blood nerve barrier in the sciatic nerves of diseased L31/CD4**^**-/- **^**mice was disrupted.** The integrity of neural barriers (BBB, BSCB and BNB) was examined with a fluorescent dye NaFlu (MW, 376 Da) injected intravenously. Quantification of NaFlu content in the brain, the spinal cord and the sciatic nerve of wild type and the symptomatic L31/CD4^-/-^ mice revealed that a significant increase of the dye within the nervous tissues was detected only in the nerve (n = 3/group, ***: p < 0.001 ) **(A)**. Histological analysis on the cross sections of the sciatic nerves confirmed the dye extravasation in the symptomatic L31 mice. Glut1 was used to label blood vessels, perinurium and epineuriums **(B)**. Scale bar: 100 μm.

The homeostasis of the nervous tissue is protected by neural barriers, from periphery to central: BBB, BSCB and BNB. As the integrity of the barriers is crucial for immune cell recruitment, we examined the functional status of the barriers with a fluorescent tracer, NaFlu. Compared with intact sciatic nerves from wild type mice, the content of NaFlu in diseased sciatic nerves was dramatically increased (Figure 6A-B), suggesting a disruption of the BNB. Interestingly, the levels of NaFlu in the brain and the spinal cord of symptomatic mice remained similar to those of wild type mice (Figure 6A), further confirming the pathology was primarily in the PNS.

### Focal inflammatory reaction in the DRG and the roots of pre-symptomatic L31/CD4^-/-^ mice, preceding disease onset

**Figure 7:**
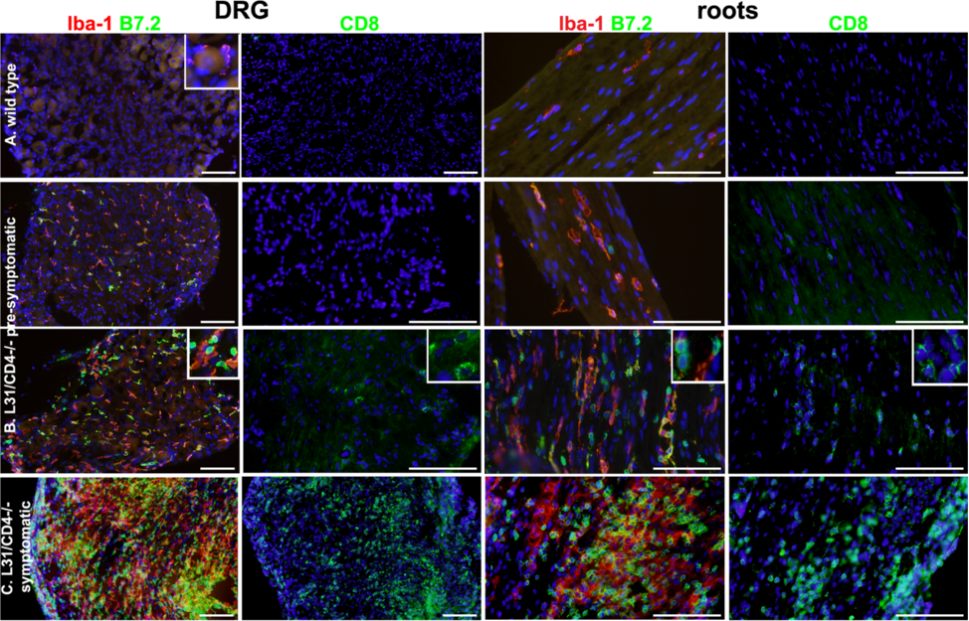
**Focal inflammatory reaction appeared in the DRG and the roots of pre-symptomatic L31/CD4**^**-/- **^**mice, preceding the disease onset.** While there were only few resident macrophages and no CD8^+^ T cells in the DRG and the roots of wild type mice **(A)**, both types of immune cells were found in these two organs in pre-symptomatic L31/CD4^-/-^ mice **(B)**. Some mice showed a slightest degree of infiltration, presumably the earliest pathologic changes **(B-upper panels)**, some had significant amount of both Iba1^+^ macrophages and CD8^+^ T cells with increased B7.2 expression **(B-bottom panels)** suggesting the status close to the disease onset **(B)**. In symptomatic L31/CD4^-/-^ mice, the DRG and the roots were fulfilled with infiltrated Iba1^+^ macrophages and CD8^+^ T cells. Both expressed high levels of B7.2 **(C)**. Scale bar: 100 μm.

If the BNB restricts the entrance of circulating immune cells into the nerves, then vessels at 3 places of the PNS, including spinal roots, DRG and nerve terminals, are physiologically permeable to various components in the circulation, where most immune cells could definitely have direct access to the endoneurium. While there were some resident Iba-1^+^ macrophages and no CD8^+^ T cells in the DRG and the roots of wild type mice (Figure 7A), immune infiltrates were apparent in these neural structures of pre-symptomatic animals (Figure 7B), which was not the case in sciatic nerves, nor in spinal cords. It was possible to detect the slightest degree of infiltration, presumably the earliest pathologic changes (Figure 7B, upper panels) and also significant amount of both macrophages and CD8^+^ T cells with high levels of B7.2 expression (Figure 7B, bottom panels) suggesting a status close to disease onset. Not surprisingly, the DRG and roots of symptomatic mice were virtually flooded with both macrophages and CD8^+^ T cells, where DRG sensory neurons were barely observable (Figure 7C).

## Discussion

We report here that mice that have been genetically modified to have an increased expression of co-stimulator B7.2 on APCs of the nervous system developed spontaneous autoimmune peripheral polyneuropathy. Pathological changes were characterized by immune cell infiltration, demyelination and axonal damage in peripheral nerves, with restricted damage in spinal cords. The infiltration of immune cells was initiated, prior to the disease onset, in the DRG and the spinal roots where the BNB is physiologically fenestrated.

### L31/CD4^-/-^ mice mimic clinical and pathological aspects of GBS in human

The most common initial symptoms of GBS are numbness, paresthesia, weakness and pain in the limbs or some combination of these symptoms. The main feature is progressive bilateral and relatively symmetric weakness of the limbs, and the weakness progresses over a period of 12 hours to 28 days before a plateau is reached [20]. Our results demonstrated that L31/CD4^-/-^ mice replicated many clinical aspects of this complex human autoimmune disease, including both sensory and motor deficits, and a temporal course of an acute onset and a stable progression [21-23]. L31/CD4^-/-^ mice also imitated pathological changes in GBS, with characteristic lymphocytes and macrophages-associated demyelination and axonal damage at the peripheral nerves, suggesting these mice might represent more closely axonal variants of GBS. Although it remains to be determined what are the specific antigens (myelin or axon or both) for autoimmune response, demyelination in peripheral nerves of L31/CD4^-/-^ mice is still distinguishable with myelin loss secondary to Wallerian degeneration by the massive infiltration of T cells which was initiated prior to the damage of the nervous tissues. Comparable to what we observed restricted damage in spinal cords, loss of myelinated fibers in posterior tracts or inflammatory infiltrates/spinal microglia activation in the grey matter have been reported in human GBS [24,25]. Similar neurological deficits and pathological changes in peripheral nerves were also detected in L31 mice (data not shown). The L31/CD4^-/-^ mouse model had many similarities with the EAN [3,26], one of the essential tools in the research of autoimmune demyelinating diseases of the PNS. However the use of EAN was restricted due to the administration of powerful immune adjuvants to break-down self-tolerance. The field lacks of spontaneous and translatable models for mechanistic studies. The impressive resemblance between the L31/CD4^-/-^ mouse model and human GBS implies it as an unequivocal experimental setting for spontaneous autoimmune peripheral polyneuropathy, especially as it allows taking advantage of the wealth of genetic resources on the C57BL/6 background.

### L31/CD4^-/-^ model provides genetic background for deciphering molecular and cellular mechanisms in GBS

Co-stimulatory signals play a key role in regulating T cell activation and are believed to have a decisive influence in the induction and penetration of cellular effecter mechanisms in spontaneous autoimmune peripheral polyneuropathy. Sensitization of APCs by increasing the expression of B7.2 through genetic approaches simulates certain common pre-dispositive conditions in GBS, e.g., infection, which is usually the most potent stimulus for B7 expression [27,28]. T cells are required in the pathogenic sequence of immune-mediated nerve damage [29], but the relative contributions of CD4^+^ and CD8^+^ T cells are controversial. Although most inflammatory infiltrates in the PNS of rats with EAN are CD4^+^ cells [30], further studies indicated that CD8^+^ cells may also be involved in the pathogenesis [31]. The necessity of both CD4^+^ and CD8^+^ cells in the genesis of EAN was confirmed by using CD4 and CD8 knock-out mice, where the severity of clinical scores and histopathological manisfestations of P0 peptide induced EAN was significantly lower than those in their wild type counterparts [32]. In human GBS, very limited *post mortem* and biopsy data is available. However, one autopsy study provided clear evidence for a potential involvement of cytotoxic T cells in demyelination. Wanschitz et al [33] reported that in all 11 examined subjects, among infiltrated endoneurial T cells, the ratio of CD8^+^ to CD3^+^ was around 0.7 to 1.4. The density of CD8/CD3 double positive T cells increased significantly in cases with more than 4 weeks duration of illness. Many of these CD8^+^ cells were found granzyme B positive; Schwann cells and myelin sheaths were detected with upregulation of MHC class I molecules [33]. In coincidence with the observation in GBS patients, our previous and current studies with L31 and L31/CD4^-/-^ mice demonstrated that 1) altering co-stimulation signals primed APCs in the nervous tissue mice and drove a predominately CD8^+^ T cell mediated autoimmune reaction [12], 2) CD8^+^ cells are able to lead to demyelination in the peripheral nerves if they can find co-stimulation in the PNS; 3) The demyelinating disease can be initiated in the absence of CD4^+^ T cells, even displaying accelerated disease development and increased expressivity [13], which indicates an immunoregulatory role for CD4^+^ T cells in disease pathogenesis. Hence, L31 and L31/CD4^-/-^ mice are unique to understand molecular pathways by which CD8^+^ T cells are involved in demyelination and axonal damage. Albeit, a characterization of functional phenotypes of CD8^+^ cells in the demyelinating nerves at different stages of the disease is essential, which will provide insights to understand the roles of CD8^+^ cells in the pathogenesis. Furthermore, crucial questions with regard to the decisive contribution of macrophages, as well as interaction between macrophages and lymphocytes in the context of demyelination and axonal loss could be explored in L31 and L31/CD4^-/-^ mice. However, whether and to what extent antibody mediated humoral immunity is involved in the disease genesis of L31 and L31/CD4^-/-^ mice need to be determined in future experiments.

### Limitation of L31 and L31/CD4^-/-^ mice as a model for human GBS

Most patients with GBS recover spontaneously, 62% had made a complete or almost complete recovery at one year [34]. However, neurological disorders in either L31 [12] or L31/CD4^-/-^ mice, with a sudden onset, seem to persist and remain stable. Whether severe myelin/axonal damage and absence of overt recovery in L31/CD4^-/-^ and L31 mice could be attributed to the lack of CD4^+^ T cell population in the symptomatic nerves is an intriguing topic for further investigation. Or it might be due to the fact that this model reflects more closely to axonal variants of GBS, since most AMAN patients have more delayed recovery than AIDP [35] and ASMAN has been associated with a more severe course and poorer prognosis [36].

### Proposed cascade of autoimmune demyelination in L31/CD4^-/-^ (and L31) model: implication for GBS

It remains presently elusive how the cascade of autoimmune responses targeting PNS structures is ignited. The fact that infiltration of immune cells was initiated before disease onset, in the roots and the DRG where the BNB is virtually absent [37] led us to propose the following cascade: 1) Transgene or infection derived- over-expression of co-stimulator B7.2 sensitizes APCs in the nervous tissue; 2) T cells first access the nervous tissue during immune surveillance through the DRGs and the roots where the endoneurial vasculature is basically permeable to various components in the blood stream; 3) T cells, especially CD8^+^ T cells that have specificities for PNS self-antigens presented by primed macrophages are engaged in productive antigen recognition and are reactivated due to the high levels of B7.2; 4) In aid with massive infiltration of macrophages, CD8^+^ T cells exert their cytotoxic effector functions, leading to destructive autoimmunity; 5) CD4^+^ T cells, could in fact, exert immunoregulatory functions during the disease course.

## Conclusion

In conclusion, with constitutive expression of B7.2 in APCs of the nervous tissues, L31/CD4^-/-^ (and L31) mice mimic many clinical and pathological signatures of GBS in human and provides an unconventional opportunity to experimentally explore the critical events that lead to spontaneous, autoimmune demyelinating disease of the PNS.

## Competing interests

The authors declare that they have no competing interests.

## Authors’ contributions

MY, SF and JZ conceived, designed the study, and drafted the manuscript; MY, AR and XQS performed experiments; all authors participated in analyzing data. All authors read and approved the final manuscript.

## Supplementary Material

Additional file 1: Table S1 List of instruments and reagents used in the study. Click here for file

Additional file 2: Figure S1 Representative experiment of B7.2 expression in the nervous tissues using flow cytometry analysis. Single-cell suspension was prepared from sciatic nerves (A) and spinal cords (B) of wild type and pre-symptomatic L31/CD4^-/-^ mice. Number of macrophages (CD11b^+^CD45^+^) in the nerves and microglia (CD11b^+^CD45^+^) in the spinal cords was counted among 2 × 10^4^ cells isolated from each tissue sample (upper panels). Histograms for B7.2 expression were gated on CD11b^+^CD45^+^ cells (lower panels). Note that compared with wild type mice, B7.2 expression level in L31/CD4^-/-^ transgenic mice is up-regulated in the nerves (6.13% vs 1.33%) as well as in the spinal cords (44.30% vs 0%). The number of macrophages and microglia also increased before the onset of the disease, 3.40% vs 0.46% and 1.92% vs 1.13%, respectively. Click here for file

Additional file 3: Video 1 and 2 L31/CD4^-/-^ mice developed motor deficits. When lifted by tail, B7.2 L31 Tg (L31/CD4^-/-^) mouse had difficulty to struggle, it remained in clasping position, suggesting weakness of both hind limbs (score 3) (video 1). B7.2 L31 Tg (L31/CD4^-/-^) mouse showed a limp tail with reduced tonus (score 1), while wild type mouse can easily elevate the tail (video 2). B7.2 L31 Tg (L31/CD4^-/-^) mouse had difficulty to walk properly and failed to support body weight when standing. Right hind limb of B7.2 L31 Tg (L31/CD4^-/-^) mouse stayed in splaying position, indicating paresis of right hind limb (score 4) (Video 2). Click here for file

Additional file 4: Figure S2 Representative experiment of macrophages and CD8^+^ T cell infiltration in the sciatic nerves with flow cytometry analysis 2 × 10^4^ cells isolated from the sciatic nerves of either wild type, or L31/CD4^-/-^ pre-symptomatic or symptomatic mice were stained with anti-CD45, anti-CD11b, anti-CD8α antibodies. Histograms for macrophages (CD11b^+^) were gated on CD45^+^ cells (upper panels). Histograms for CD8^+^ T cells were gated on CD45^+^ cells (lower panels). A dramatic increase of CD11b^+^CD45^+^ macrophages and CD8^+^CD45^+^ T cells were found in symptomatic L31/CD4^-/-^ mice, while increase of immune cells in pre-symptomatic L31/CD4^-/-^ mouse sciatic nerves was almost undetectable or moderate. Click here for file

Additional file 5: Figure S3 Representative experiment of microglia and CD8^+^ T cell infiltration in the lumbar spinal cords with flow cytometry analysis 2 × 10^4^ cells isolated from the lumbar spinal cords of either wild type, or L31/CD4^-/-^ pre-symptomatic or symptomatic mice were stained with anti-CD45, anti-CD11b, anti-CD8α antibodies. Histograms for microglia (CD11b^+^) were gated on CD45^+^ cells (upper panels). Histograms for CD8^+^ T cells were gated on CD45^+^ cells (lower panels). A significant increase of CD11b^+^CD45^+^ microglia and CD8^+^CD45^+^ T cells were detected in symptomatic L31/CD4^-/-^ mouse spinal cords, with a slight change in pre-symptomatic mice. Click here for file
