# Supplementary material for: A new animal model of spontaneous autoimmune peripheral polyneuropathy: implications for Guillain-Barré syndrome
Source: Acta Neuropathol Commun. 2014 Jan 8;2:5. doi: 10.1186/2051-5960-2-5 (PMC3895684; doi:10.1186/2051-5960-2-5)
Supplement: Additional file 1: Table S1 — List of instruments and reagents used in the study. [file 2051-5960-2-5-S1.docx]

| **Reagents** |  |
| --- | --- |
| Acetone | Fisher BioReagents (Fair Lawn, NJ) |
| Paraformaldehyde | Electron Microscopy Sciences (Hatfield, PA) |
| 0.05 M sodium phosphate buffer | GIBCO, Invitrogen (Grand Island, NY) |
| Ethylene glycol | Fisher BioReagents (Fair Lawn, NJ) |
| Glycerol | Fisher BioReagents (Fair Lawn, NJ) |
| Isoflurane | Baxter Corporation (Mississauga, ON) |
| RPMI-1640 medium | GIBCO, Invitrogen (Grand Island, NY) |
| Penicillin-Streptomycin | GIBCO, Invitrogen (Grand Island, NY) |
| Fetal bovine serum | GIBCO, Invitrogen (Grand Island, NY) |
| Trizma base | Sigma-Aldrich (St. Louis, MO) |
| Sodium chloride | Sigma-Aldrich (St. Louis, MO) |
| Triton-X100 | Sigma-Aldrich (St. Louis, MO) |
| Bovine serum albumin | HyClone (South Logan, UT) |
| Normal goat serum | Vector Laboratories (Burlingame, CA) |
| Vectashield mounting medium | Vector Laboratories (Burlingame, CA) |
| NaFlu; MW, 376 | Sigma-Aldrich (St. Louis, MO) |
| CompBeads | BD Biosciences (San Diego, CA) |
|  |  |
| **Instruments** |  |
| Rotarod assay | IITC Life Science Inc. (Woodland Hills, CA) |
| Calibrated monofilaments | Stoelting Co. (Wood Dale, IL) |
| Hot plate | Biological Research Apparatus (Comerio, Italy) |
| Cryostat  Microtome | LEICA Microsystem (Nussloch, Germany)  LEICA Microsystem (Nussloch, Germany) |
| Olympus BX51 | Olympus (Tokyo, Japan) |
| Olympus DP71 | Olympus (Tokyo, Japan) |
| Fluoview 1000 | Olympus (Tokyo, Japan) |
| LSR Fortessa flow cytometer | BD Biosciences (San Jose, CA |
| Flow Jo software | Tree star Inc. (Ashland, OR) |
| Spectrophotofluorometer | Molecular Devices (Orleans Drive Sunnyvale, CA) |
