# Supplementary figures and images for: A new animal model of spontaneous autoimmune peripheral polyneuropathy: implications for Guillain-Barré syndrome
Source: Acta Neuropathol Commun. 2014 Jan 8;2:5. doi: 10.1186/2051-5960-2-5 (PMC3895684; doi:10.1186/2051-5960-2-5)

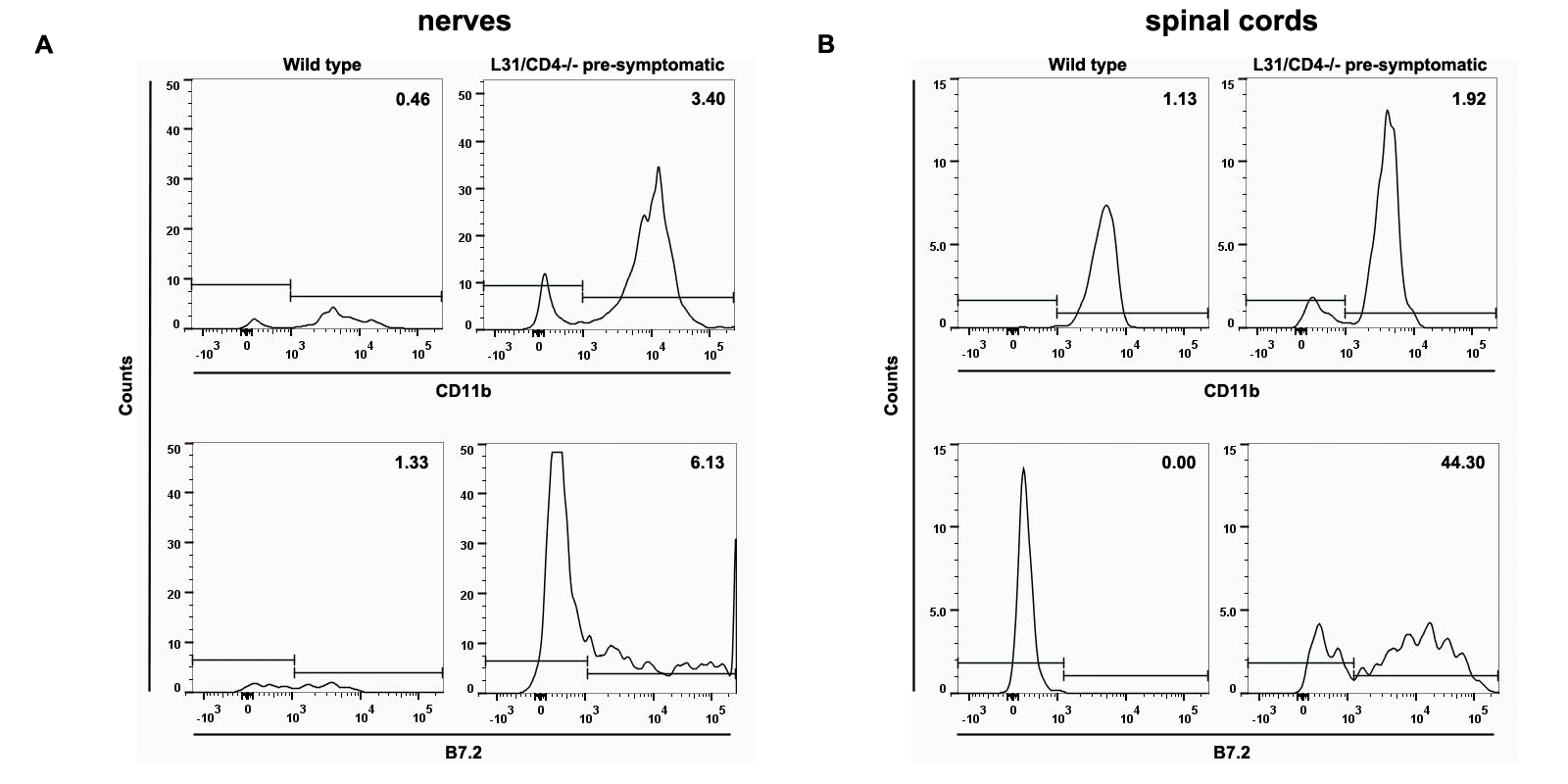

Supplement: Additional file 2: Figure S1 — Representative experiment of B7.2 expression in the nervous tissues using flow cytometry analysis. Single-cell suspension was prepared from sciatic nerves (A) and spinal cords (B) of wild type and pre-symptomatic L31/CD4-/- mice. Number of macrophages (CD11b+CD45+) in the nerves and microglia (CD11b+CD45+) in the spinal cords was counted among 2 × 10^4^ cells isolated from each tissue sample (upper panels). Histograms for B7.2 expression were gated on CD11b+CD45+ cells (lower panels). Note that compared with wild type mice, B7.2 expression level in L31/CD4-/- transgenic mice is up-regulated in the nerves (6.13% vs 1.33%) as well as in the spinal cords (44.30% vs 0%). The number of macrophages and microglia also increased before the onset of the disease, 3.40% vs 0.46% and 1.92% vs 1.13%, respectively. [file 2051-5960-2-5-S2.tiff]

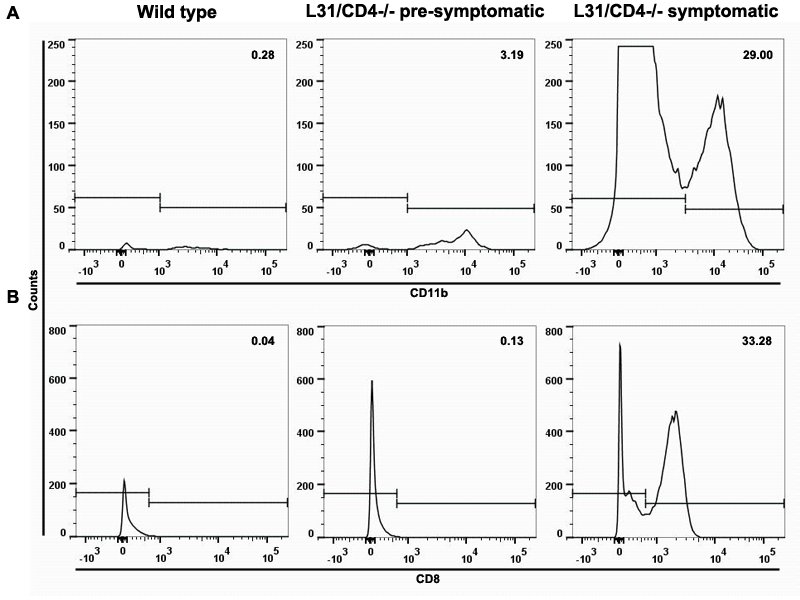

Supplement: Additional file 4: Figure S2 — Representative experiment of macrophages and CD8+ T cell infiltration in the sciatic nerves with flow cytometry analysis 2 × 10^4^ cells isolated from the sciatic nerves of either wild type, or L31/CD4-/- pre-symptomatic or symptomatic mice were stained with anti-CD45, anti-CD11b, anti-CD8α antibodies. Histograms for macrophages (CD11b+) were gated on CD45+ cells (upper panels). Histograms for CD8+ T cells were gated on CD45+ cells (lower panels). A dramatic increase of CD11b+CD45+ macrophages and CD8+CD45+ T cells were found in symptomatic L31/CD4-/- mice, while increase of immune cells in pre-symptomatic L31/CD4-/- mouse sciatic nerves was almost undetectable or moderate. [file 2051-5960-2-5-S4.tiff]

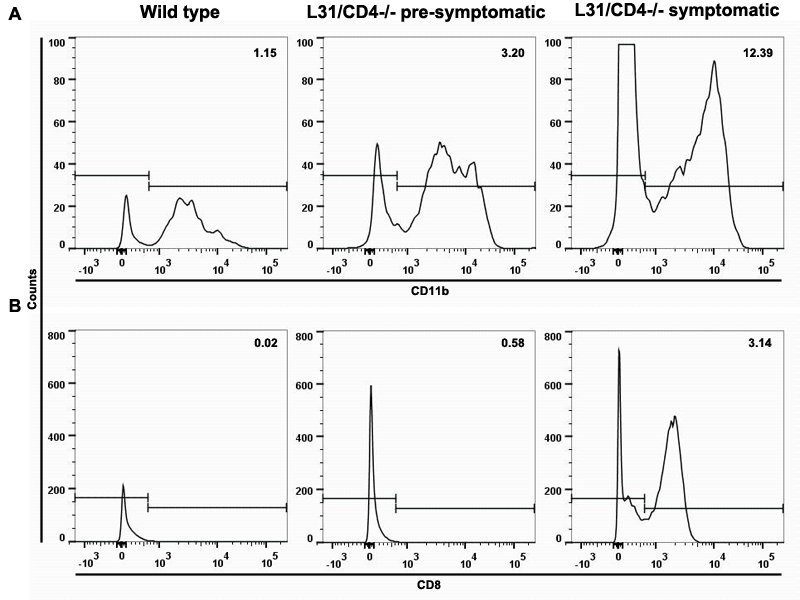

Supplement: Additional file 5: Figure S3 — Representative experiment of microglia and CD8+ T cell infiltration in the lumbar spinal cords with flow cytometry analysis 2 × 10^4^ cells isolated from the lumbar spinal cords of either wild type, or L31/CD4-/- pre-symptomatic or symptomatic mice were stained with anti-CD45, anti-CD11b, anti-CD8α antibodies. Histograms for microglia (CD11b+) were gated on CD45+ cells (upper panels). Histograms for CD8+ T cells were gated on CD45+ cells (lower panels). A significant increase of CD11b+CD45+ microglia and CD8+CD45+ T cells were detected in symptomatic L31/CD4-/- mouse spinal cords, with a slight change in pre-symptomatic mice. [file 2051-5960-2-5-S5.tiff]
